# Using a Clinicopathologic and Gene Expression (CP-GEP) Model to Identify Stage I–II Melanoma Patients at Risk of Disease Relapse

**DOI:** 10.3390/cancers14122854

**Published:** 2022-06-09

**Authors:** Evalyn E. A. P. Mulder, Iva Johansson, Dirk J. Grünhagen, Dennie Tempel, Barbara Rentroia-Pacheco, Jvalini T. Dwarkasing, Daniëlle Verver, Antien L. Mooyaart, Astrid A. M. van der Veldt, Marlies Wakkee, Tamar E. C. Nijsten, Cornelis Verhoef, Jan Mattsson, Lars Ny, Loes M. Hollestein, Roger Olofsson Bagge

**Affiliations:** 1 Departments of Surgical Oncology, Erasmus MC Cancer Institute, 3015 GD Rotterdam, The Netherlands; e.e.a.p.mulder@erasmusmc.nl (E.E.A.P.M.); d.grunhagen@erasmusmc.nl (D.J.G.); d.verver@erasmusmc.nl (D.V.); c.verhoef@erasmusmc.nl (C.V.); 2 Departments of Medical Oncology, Erasmus MC Cancer Institute, 3015 GD Rotterdam, The Netherlands; a.vanderveldt@erasmusmc.nl; 3 Departments of Pathology, Sahlgrenska University Hospital, 413 45 Gothenburg, Sweden; iva.johansson@gu.se; 4 Departments of Oncology, Institute of Clinical Sciences at Sahlgrenska Academy, Gothenburg University, 405 30 Gothenburg, Sweden; lars.ny@oncology.gu.se; 5 SkylineDx B.V., 3062 ME Rotterdam, The Netherlands; d.tempel@skylinedx.com (D.T.); b.rentroia@skylinedx.com (B.R.-P.); j.dwarkasing@skylinedx.com (J.T.D.); 6 Department of Pathology, Erasmus MC Cancer Institute, 3015 GD Rotterdam, The Netherlands; a.mooyaart@erasmusmc.nl; 7 Departments of Radiology & Nuclear Medicine, Erasmus MC Cancer Institute, 3015 GD Rotterdam, The Netherlands; 8 Departments of Dermatology, Erasmus MC Cancer Institute, 3015 GD Rotterdam, The Netherlands; m.wakkee@erasmusmc.nl (M.W.); t.nijsten@erasmusmc.nl (T.E.C.N.); 9 Departments of Surgery, Sahlgrenska University Hospital, 413 45 Gothenburg, Sweden; bej.mattsson@telia.com (J.M.); roger.olofsson.bagge@gu.se (R.O.B.); 10 Departments of Oncology, Sahlgrenska University Hospital, 413 45 Gothenburg, Sweden; 11 Department of Research, Netherlands Comprehensive Cancer Organization (IKNL), 3511 DT Utrecht, The Netherlands; 12 Departments of Surgery, Institute of Clinical Sciences at Sahlgrenska Academy, Gothenburg University, 405 30 Gothenburg, Sweden; 13 Wallenberg Centre for Molecular and Translational Medicine, University of Gothenburg, 405 30 Gothenburg, Sweden

**Keywords:** primary cutaneous melanoma, clinicopathologic and gene expression model, risk stratification recurrence

## Abstract

**Simple Summary:**

More than 40% of patients initially diagnosed with ‘low risk’ (stage I–II) melanoma eventually develop melanoma recurrence or die as a result of melanoma. While the current standard of care at diagnosis for these patients is watchful waiting, they may benefit from adjuvant systemic treatment. The primary aim of this retrospective study was to assess the performance of a clinicopathologic and gene expression (CP-GEP) model, a model originally developed to predict sentinel node metastasis, to identify patients with stage I–II melanoma at risk of disease relapse. This study included Swedish and Dutch patients (18 years of age or older) with a melanoma of the skin without sentinel node metastasis. Using the non-invasive CP-GEP model, the patients could be divided into two groups: high (413 patients) or low risk (122 patients) of recurrence. While these results are promising, optimization of the CP-GEP model is recommended before implementing the model in clinical practice.

**Abstract:**

Background: The current standard of care for patients without sentinel node (SN) metastasis (i.e., stage I–II melanoma) is watchful waiting, while >40% of patients with stage IB–IIC will eventually present with disease recurrence or die as a result of melanoma. With the prospect of adjuvant therapeutic options for patients with a negative SN, we assessed the performance of a clinicopathologic and gene expression (CP-GEP) model, a model originally developed to predict SN metastasis, to identify patients with stage I–II melanoma at risk of disease relapse. Methods: This study included patients with cutaneous melanoma ≥18 years of age with a negative SN between October 2006 and December 2017 at the Sahlgrenska University Hospital (Sweden) and Erasmus MC Cancer Institute (The Netherlands). According to the CP-GEP model, which can be applied to the primary melanoma tissue, the patients were stratified into high or low risk of recurrence. The primary aim was to assess the 5-year recurrence-free survival (RFS) of low- and high-risk CP-GEP. A secondary aim was to compare the CP-GEP model with the EORTC nomogram, a model based on clinicopathological variables only. Results: In total, 535 patients (stage I–II) were included. CP-GEP stratification among these patients resulted in a 5-year RFS of 92.9% (95% confidence interval (CI): 86.4–96.4) in CP-GEP low-risk patients (*n* = 122) versus 80.7% (95%CI: 76.3–84.3) in CP-GEP high-risk patients (*n* = 413; hazard ratio 2.93 (95%CI: 1.41–6.09), *p* < 0.004). According to the EORTC nomogram, 25% of the patients were classified as having a ‘low risk’ of recurrence (96.8% 5-year RFS (95%CI 91.6–98.8), *n =* 130), 49% as ‘intermediate risk’ (88.4% 5-year RFS (95%CI 83.6–91.8), *n =* 261), and 26% as ‘high risk’ (61.1% 5-year RFS (95%CI 51.9–69.1), *n =* 137). Conclusion: In these two independent European cohorts, the CP-GEP model was able to stratify patients with stage I–II melanoma into two groups differentiated by RFS.

## 1. Introduction

More than 40% of patients initially diagnosed with early-stage melanoma (AJCC stage I–II) will present with disease recurrence or die as a result of melanoma [1,2]. Although the 5-year melanoma-specific survival (MSS) rates in patients with stage IIA–C are comparable to those for IIIA/B melanoma (93–83% versus 94–82%) [3], the strategy after surgery is different. Despite the equivalent risk of recurrence, the current standard of care for patients without (sentinel node) SN metastasis is watchful waiting, whereas patients with resected stage III melanoma are currently eligible for adjuvant systemic treatment [4,5,6].

Adjuvant treatment with immune checkpoint inhibitors (anti-programmed death-1 (anti-PD-1), nivolumab, or pembrolizumab) for patients with stage III melanoma has significantly improved the recurrence-free survival (RFS) rates, while data on overall survival (OS) are still eagerly awaited. Recently, the first results on adjuvant treatment for patients with stage IIB/C melanoma with the PD1-inhibitor pembrolizumab versus a placebo were presented [7]. The 12-month interim analysis of this KEYNOTE-716 trial showed a reduced recurrence rate in the patients receiving pembrolizumab compared to a placebo (11.1% versus 16.8%). Based on these first results, the FDA has recently approved pembrolizumab for the adjuvant treatment of stage IIB and IIC melanoma, and more adjuvant systemic treatments are expected for stage II melanoma. However, immune checkpoint inhibitors can cause severe and durable immune-related toxicities [8], which is different from adjuvant treatment with targeted therapies (BRAF/MEK inhibitors, e.g., dabrafenib–trametinib) with which most patients only experience adverse events during treatment [9]. In addition, all these systemic treatments have a considerable impact on healthcare budgets [10,11]. To prevent overtreatment in patients who can be cured by surgery alone, new diagnostic tools are needed to identify patients with early-stage melanoma who are at risk of disease recurrence and may benefit from adjuvant systemic treatment.

Several tools have been developed to identify patients with melanoma at high risk of disease recurrence or metastasis, thereby focusing on clinicopathological (CP) features [12,13,14,15] and/or gene expression profiles (GEP). One of these tools is the European Organization for Research and Treatment of Cancer (EORTC) nomogram. This nomogram is based on CP features (i.e., Breslow thickness, ulceration, anatomical location of the primary melanoma, and a negative SN) and has been validated in a large European cohort to predict recurrence and melanoma-specific mortality for patients with a negative SLNB [12].

In addition to clinical and pathological variables, a number of GEPs have been developed to identify patients at high risk of disease recurrence [16,17,18]. Recently, a clinicopathologic and gene expression (CP-GEP) model, which combines patients’ age and Breslow thickness at primary diagnosis with the expression of eight genes (ITGB3, PLAT, SERPINE2, GDF15, TGFBR1, LOXL4, CXCL8, and MELANA), has been developed and validated to predict a low risk of SN metastasis [19,20]. These genes were identified from 16,029 sequenced genes. For a detailed methodology of this model development, we refer to Meves et al. and the development paper by Bellomo et al. [19,21]. Since this tool can be applied to resected primary melanoma, sentinel node biopsies can be omitted in patients with a low risk of SN metastasis. In addition, the CP-GEP model was able to identify patients with stage I–IIA melanoma and a high risk of disease recurrence in a US cohort (*n =* 837) [16,22]. The primary aim of the current study was to investigate whether the CP-GEP model could identify patients with stage I–II melanoma and a high risk of recurrence based on two independent European patient cohorts. The secondary aim was to compare the results of the CP-GEP model to a model with only CP variables. 

## 2. Patients and Methods

### 2.1. Study Population

Patients with cutaneous melanoma aged ≥18 years and with a negative SLNB at the Sahlgrenska University Hospital (between October 2006 and December 2014) and Erasmus MC Cancer Institute (between January 2007 and December 2017) were included. Patients with multiple primary melanomas prior to SLNB, missing data on age or Breslow thickness, missing formalin-fixed paraffin-embedded (FFPE) tissue from the primary tumor (excised for diagnostic purpose), or an invalid quantitative polymerase chain reaction (qPCR) test were excluded [19]. Disease stages were (re)staged according to the AJCC 8th edition^3^.

The Swedish study was approved by the Swedish Ethical Review Authority (Dnr 908-14 and addendum 2020-00267). The Dutch study was approved by the Erasmus MC Ethics Committee (MEC-2018-1183) and the Privacy Committee of the Nationwide Network and Registry of Histopathology and Cytopathology (PALGA). Human residual tissue was used according to the code of conduct for responsible use of the Federation of Dutch Medical Scientific Societies. None of the patients objected to the use of their residual tissue for scientific research.

### 2.2. CP-GEP Model

The CP-GEP combines clinical (patient’s age) and pathological (Breslow thickness) features with the gene expression in the primary melanoma of eight target genes (ITGB3, PLAT, SERPINE2, GDF15, TGFBR1, LOXL4, CXCL8, and MLANA) corrected by the mean of two housekeeping genes (RLP0 and ACTB) using the ΔCt method [19]. For each patient, the CP-GEP score was compared with a predefined predicted probability cut-off value of 0.063, resulting in a binary outcome: CP-GEP low risk and CP-GEP high risk.

Samples from Sweden were identified in a prospective database for all patients undergoing SLNB. The FFPE blocks from each primary tumor were retrieved from the pathology archives. After initial trimming, two 4 µm sections for routine hematoxylin and eosin (H&E) staining and one 50 µm section for RNA extraction per tumor were obtained from the blocks with the deepest presentation and largest volume of invasive melanoma. Cutting was performed on a rotary microtome under RNase free conditions. In order to prevent RNA contamination between the patients, the microtome knife was changed for every patient and the microtome was cleaned.

Samples from The Netherlands were identified by linking Erasmus MC patient files to PALGA. The FFPE blocks from each primary tumor were retrieved from the pathology archives and five standard-thickness (10 micron) recuts were collected in a single standard 1.5-mL microcentrifuge tube and stored refrigerated until RNA isolation. Details on the retrieval of samples in The Netherlands were described earlier [22].

Dedicated melanoma pathologists at each site revised the original pathology report (from both the primary melanoma and corresponding SN). In case of discrepancy, the reviewing pathologist’s evaluation was decisive. RNA extraction and qPCR analysis were performed by SkylineDx (Rotterdam, The Netherlands). RNA isolation was performed on full-excision histological slides, without the need for macrodissection of the tumor tissue [22].

### 2.3. Outcomes

The primary endpoint of this study was 5-year recurrence-free survival (RFS). RFS was calculated from the date of primary melanoma diagnosis to the date of first metastasis, death, or last follow-up visit, whichever came first. Moreover, 10-year RFS, 5- and 10-year distant metastasis-free survival (DMFS), and 5- and 10-year overall survival (OS) were determined. DMFS was calculated from the date of primary melanoma diagnosis to the date of first distant metastasis, death, or last follow-up visit, whichever came first. Regional metastases included in-transit, satellite, and regional lymph node metastases. Distant metastases included all metastases beyond the regional lymph node basin. OS was determined from the date of primary melanoma diagnosis to the last follow-up visit or death from any cause, whichever came first.

For the Sahlgrenska University Hospital, all patients with primary cutaneous melanoma and an SLNB were registered in a prospective database, including clinical baseline data, histopathological parameters, and clinical follow-up data. The cohort included all types of cutaneous melanoma from all body regions, except melanoma of the head and neck region. Mortality data were retrieved from the Swedish Cause of Death Registry. For the Erasmus MC Cancer Institute, data on metastases (both regional and distant) were retrieved from PALGA (if histologically confirmed) or medical files (if not histologically confirmed). To determine OS, mortality data (until 1 February 2019) were obtained by linkage with The Netherlands Cancer Registry (NCR).

In addition, CP-GEP was compared with the EORTC nomogram to predict recurrence in patients with a negative SN (stage I/II) [12].

### 2.4. Statistical Analysis

A sample size calculation was not performed as all eligible patients diagnosed with stage I–II melanoma at both sites were included. To determine the prognostic value of CP-GEP, 5-year RFS, DMFS, and OS were calculated using Kaplan–Meier curves, stratified on CP-GEP output labels (low risk versus high risk). Calculation of the hazard ratio (HR) with a 95% confidence interval (CI) was completed using a Cox proportional hazard regression model, with the corresponding Wald *p*-value < 0.05 (two-sided) indicating statistical significance. The median follow-up was calculated based on reverse Kaplan–Meier estimator via R package *prodlim* (version 2019.11.13). Log–log CIs were computed for 5- and 10-year survival rate estimates. To calculate the EORTC output labels (low, intermediate, high), the EORTC nomogram, including the primary melanoma characteristics (Breslow thickness, ulceration, anatomical site), was applied to all patients in the SN-negative cohort. Patients with unknown ulceration and/or anatomical site of the primary melanoma were excluded.

Analyses were performed using R version 3.6.1 (R Core Team, Vienna, Austria; 2021). Patient characteristics were analyzed using the *gtsummary* R package (version 1.3.3). Survival analyses were performed with *survminer* (version 0.4.6) and *survival* (version 3.1.8) R packages.

## 3. Results

### 3.1. Study Population

In total, 535 stage I–II patients were included in the study, with a median age of 60 years (interquartile range (IQR) 48–70). The median Breslow thickness was 1.8 mm (IQR 1.3–3.0), and ulceration was absent in most patients (71.6%). The most reported substages were IB (42.2%) and IIA (27.7%). Superficial spreading melanoma (SSM) was the most common melanoma subtype (52.5%), and most of the melanomas were located on the trunk (47.9%), followed by the lower limbs (31.2%). Table 1 summarizes all the CP baseline characteristics, and the flowchart in Supplemental Figure S1 provides an overview of the included patients.

### 3.2. Outcomes

For all the patients, the 5-year RFS was 83.5% (95%CI: 79.9–86.5). The 5-year DMFS and 5-year OS were 88.8% (95%CI: 85.6–91.3) and 86.7% (95%CI: 83.4–89.4), respectively. From 405 (75.7%) patients, more than a 5-year follow-up was available, of which 141 (26.4%) patients were followed up to 10 years after diagnosis. In Supplementary Table S1, a detailed overview of the 5-year survival rates (RFS, DMFS, and OS) is provided per (sub)group.

**Figure 1:**
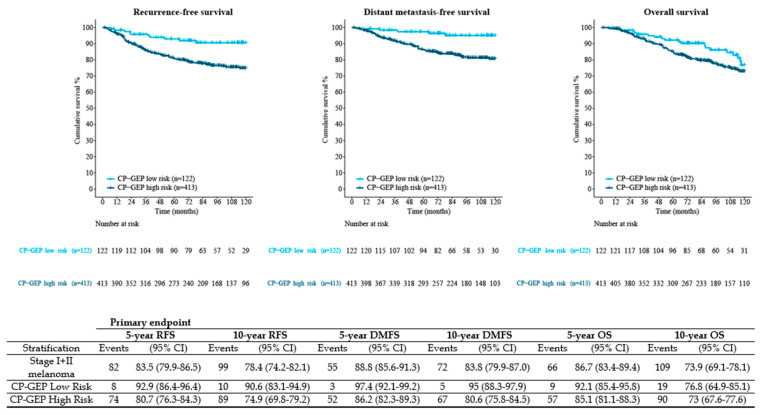
Kaplan–Meier survival curves (RFS, DMFS, and OS) stratified by CP-GEP classification. Primary endpoint is 5-year RFS. CP-GEP, clinicopathologic and gene expression profile; DMFS, distant metastasis-free survival; HR, hazard ratio; OS, overall survival; RFS, recurrence-free survival; SLNB, sentinel lymph node biopsy; 95% CI, 95% confidence interval.

For each patient, the CP-GEP risk was determined, resulting in 122 (22.8%) patients with a low-risk CP-GEP outcome and 413 (77.2%) patients with a high-risk CP-GEP outcome. To investigate the prognostic value of CP-GEP in these patients, stratification based on the CP-GEP outcome resulted in a 5-year RFS of 92.9% (95%CI: 86.4–96.4) for the patients with low-risk CP-GEP versus 80.7% (95%CI 76.3–84.3) for the patients with high-risk CP-GEP (HR 2.93, 95%CI 1.41–6.09, *p* < 0.004) (Figure 1). At 10 years, the RFS for the patients with low-risk CP-GEP was 90.6% (95%CI: 83.1–94.9) versus 74.9% (95%CI 69.8–79.2) for the patients with high-risk CP-GEP HR 2.84, 95%CI 1.47–5.45, *p* <0.002). The DMFS and OS rates are also provided in Figure 1.

**Figure 2:**
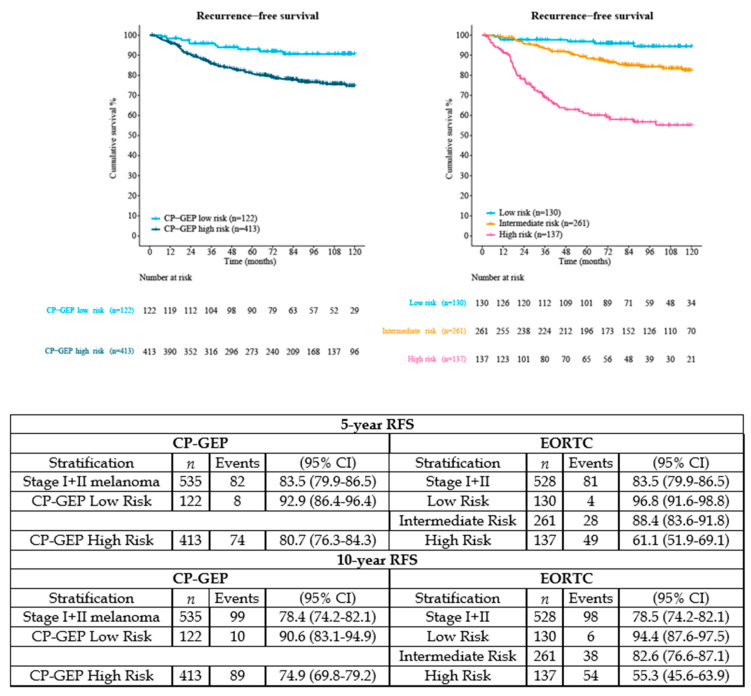
Comparison RFS between CP-GEP and EORTC nomogram in stage I–II melanoma. Because ulceration and/or anatomical site of the primary melanoma was unknown, 7 patients were excluded from the EORTC nomogram (*n =* 528 instead of *n =* 535 included for CP-GEP). Abbreviations: CP-GEP, clinicopathologic and gene expression profile; EORTC, European Organization for Research and Treatment of Cancer; RFS, recurrence-free survival; SLNB, sentinel lymph node biopsy; 95% CI, 95% confidence interval.

According to the EORTC nomogram, 25% of the patients were classified as having a ‘low risk’ of recurrence (96.8% 5-year RFS (95%CI 91.6–98.8), *n =* 130), 49% as ‘intermediate risk’ (88.4% 5-year RFS (95%CI 83.6–91.8), *n =* 261), and 26% as ‘high risk’ (61.1% 5-year RFS (95%CI 51.9–69.1), *n =* 137). In Figure 2, the RFS outcomes after double stratification (CP-GEP versus EORTC nomogram) are shown.

## 4. Discussion

In patients with stage I–II melanoma, CP-GEP was able to identify patients with a high risk of recurrence and a significantly lower 5-year RFS, DMFS, and OS. Applying both the CP-GEP risk labels (low/high) and the EORTC nomogram labels (low/intermediate/high) to our cohort, the number of patients with a low risk of recurrence appeared comparable: 22.8% (122/535) versus 24.6% (130/528) for CP-GEP versus EORTC, respectively. However, the EORTC nomogram can only be applied after an SLNB, whereas the CP-GEP could potentially be applied to resected primary melanoma. In clinical practice, it is conceivable that CP-GEP could identify patients with stage I–II melanoma at a low risk of recurrence and for whom an SLNB could be omitted.

**Figure 3:**
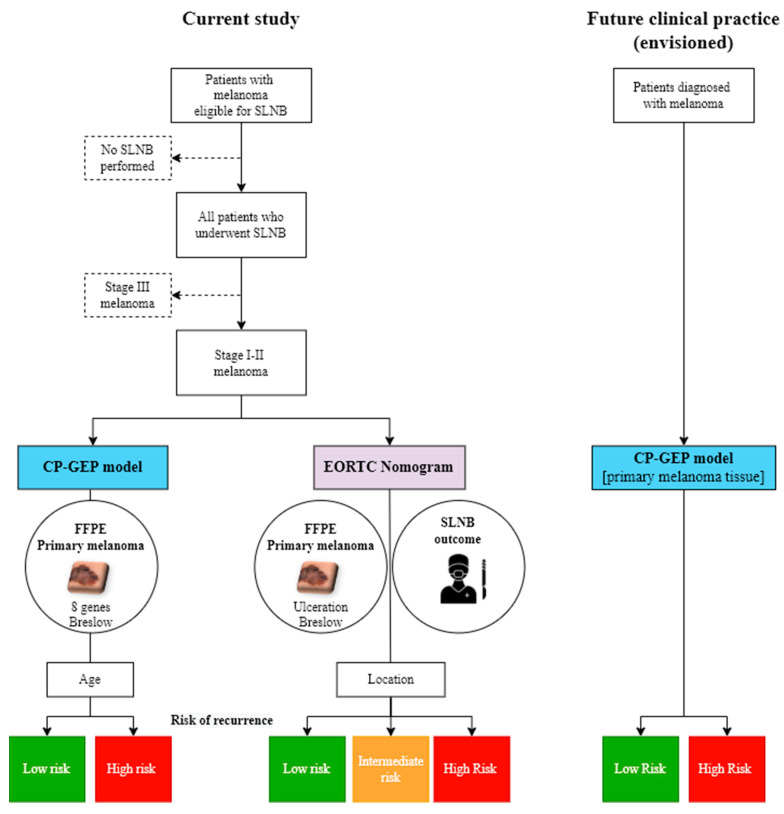
Differences in envisioned clinical practice using CP-GEP versus EORTC nomogram to predict risk of recurrence. For the application of the CP-GEP model, only tissue from the (already excised) primary melanoma is needed (in combination with Breslow depth in mm and patients’ age at diagnosis). For the EORTC nomogram, an SLNB is required. CP-GEP, clinicopathologic and gene expression profile; EORTC, European Organization for Research and Treatment of Cancer; FFPE, formalin-fixed paraffin-embedded; SLNB, sentinel lymph node biopsy; SN, sentinel node.

As a significant portion (>40%) of the patients initially diagnosed with stage I–II melanoma will present with disease recurrence or death, there is a high clinical need to identify patients with early-stage melanoma at a high risk of disease recurrence. The advantage of the EORTC nomogram is that it can be calculated for any patient since it is based on CP prognostic factors (Breslow thickness, ulceration, and anatomical location), which are almost always available. In contrast to the CP-GEP model, an SLNB outcome is required for the EORTC model [12]; to predict the risk of recurrence in patients with a positive SN, another tool is needed [23]. In contrast to the EORTC nomogram, the CP-GEP model only requires tissue from the (already excised) primary melanoma, combined with the Breslow depth in mm and patient age at diagnosis (Figure 3). A Swedish risk model based on data from the population-based Swedish Melanoma Register was also developed as a prognostic instrument for survival outcome in patients with melanoma. The combination of patient (age and gender) and tumor data (tumor site, Breslow thickness, ulceration, Clark’s level of invasion, and, when applicable, outcome of SLNB) was used to predict the 1-, 5-, and 10-year probabilities of death [15,24]. According to this model, Breslow thickness had the greatest prognostic impact; the presence of ulceration nearly doubled the risk of melanoma-specific mortality, whereas a positive SN tripled this risk.

As most people die from thin melanomas [1,2], El Sharouni and colleagues focused on identifying patients with thin (T1) melanomas at high risk of disease recurrence by developing a nomogram based on the following CP features: Breslow thickness, ulceration, melanoma subtype, tumor localization, mitosis, SN status, age, and gender [14]. Although promising, manually counting mitoses by pathologists is subjective and is often not reported (>50%, according to El Sharouni). For this reason, mitotic rate has been excluded from the AJCC 8th Staging Manual [3]. Furthermore, since the majority of SNs are negative [25,26,27,28], a prognostic model that can accurately predict survival without the need for an SLNB is required. This way, unnecessary morbidity and costs associated with this procedure can be avoided.

Thakur et al. identified a novel transcriptomic signature of primary melanoma with a similar prognostic value to the AJCC staging but with added prognostic value in stage I melanoma [29]. Its predictive value was also tested in published data from patients with metastatic melanoma receiving immunotherapy; however, this signature has not yet been externally validated. The 31-GEP assay (twenty-eight discriminating, three control genes, DecisionDx-Melanoma) by Gerami et al. has investigated the metastatic risk most extensively and has also included thin melanomas [17]. In patients with stage I and II melanoma, the 31-GEP assay was a significant predictor of the RFS and DMFS in a multivariable model. Although this 31-GEP has been investigated in several studies [30,31,32,33,34], its clinical utility in thin melanomas is under debate [35]. Another test is the 11-GEP (MelaGenix) assay [16]. A recent systematic review and meta-analysis examining the performance of the 31-GEP and a 11-GEP assays showed limited prognostic potential of both assays in stage I disease [36]. Another GEP-predicting outcome (progression-free survival and OS) in patients with melanoma used transcriptomic data from the phase III adjuvant AVAST-M study (Cam_121 signature) [18]. Although the AVAST-M study only included patients with stage IIB–III melanoma, the Cam_121 signature was externally validated within the cohort from Thakur (also including stage I/IIA patients), amongst others. This validation confirmed that Cam_121 was associated with MSS in both univariate and multivariate Cox regression models [18].

The CP-GEP model was specifically developed as a rule-out test by having a high negative predictive value to identify patients that may forgo an SLNB [19]. To use the CP-GEP as a rule-in test to predict survival and guide clinical decisions, the operating point must be optimized to identify patients at high risk of disease recurrence; the aim is to achieve a high positive predictive value, avoiding under-staging of patients who might be eligible for adjuvant treatment. In fact, the current operating point seemed too conservative as most of the patients with stage I–II melanoma were labeled as being at high risk of disease recurrence. Furthermore, additional variables (either clinical, pathological, and/or gene expression) may be required to accurately predict the outcome.

One of the strengths of this study is that all the consecutive patients who underwent an SLNB could be included without being subjected to additional procedures. Combining the Swedish and Dutch patient cohorts increased the representativeness of a European population and strengthened the external validity of the results. By linking the dataset with Swedish and Dutch registries that report death, follow-up data for survival were complete.

While the results of the CP-GEP and other CP and/or GEP-based tools seem promising, there are some limitations that hinder clinical implementation. Due to the sample size and the small number of events within stage I melanoma, we were unable to stratify the analyses according to stage and gene expression analysis, which is not (yet) performed routinely. Therefore, neither this tool nor other (GEP) tools are currently included in the NCCN guidelines^®^ for melanoma [37]. To clearly demonstrate the clinical utility for individualized treatment, new prediction tools should be tested and validated in independent datasets. Furthermore, a consensus must be reached on which patients are eligible for adjuvant treatment. Following the positive and robust data on RFS from the pivotal phase III trials for both anti-PD-1 (CheckMate 238 and KEYNOTE-054) and BRAF/MEK inhibitors (COMBI-AD) [38,39,40], patients with resected stage III (lymph node metastasis >1 mm) are currently eligible for adjuvant systemic treatment (i.e., after complete surgical resection). Since the MSS rates in patients with stage II melanoma are comparable to the MSS rates in patients with IIIA/B melanoma without adjuvant therapy [3], there is an ongoing debate as to whether these high-risk melanoma patients should also be treated in the adjuvant setting. In line with this debate, the recent positive data from the KEYNOTE-716 trial showed a significantly prolonged RFS at 12 months in patients with stage IIB/C melanoma treated with pembrolizumab (90.5%) versus a placebo (83.1%) in the adjuvant setting (HR 0.65, 95% CI 0.46–0.92; *p* = 0.00658; median not reached for both) [7]. These recent developments underscore the need for new tools to identify patients with stage I–II melanoma at a high risk of recurrence. We showed that risk prediction based on genetic information in the primary melanoma combined with CP variables is promising. However, in its present form, the CP-GEP model is not yet suitable for clinical practice as the current false positive rate is too high to select patients for adjuvant therapy and would result in overtreatment. To prevent overtreatment, while keeping healthcare affordable, benefits and costs should also be considered, especially since adjuvant systemic therapies are expected to be introduced in the near future for patients with non-metastatic (i.e., SN-negative) melanoma.

## 5. Conclusions

The CP-GEP model is a promising non-invasive tool that can be used to stratify patients with cutaneous melanoma into categories of high or low risk of disease recurrence. CP-GEP is able to identify patients with stage I–II melanoma that have a high risk of recurrence based on the primary melanoma. Since the model was originally developed to predict SN metastasis, optimization of the model for specific patient subgroups would be needed. By doing so, it can be optimized to predict recurrence in stage I–II melanoma with high accuracy to prevent overtreatment and identify patients who will benefit from adjuvant treatment.

## Figures and Tables

**Table 1:** Baseline characteristics of patients with stage I–II melanoma. *n* (%) or median (interquartile range). ALM, acral lentiginous melanoma; LMM, lentigo maligna melanoma; NM, nodular melanoma; SLNB, sentinel lymph node biopsy; SSM, superficial spreading melanoma. * According to AJCC 8.

Variable		Sahlgrenska University Hospital *n* = 367	Erasmus MC Cancer Institute *n* = 168	Combined *n* = 535
Gender	Female	181 (49.3%)	80 (47.6%)	261 (48.8%)
	Male	186 (50.7%)	87 (51.8%)	273 (51.0%)
	Unknown	0 (0%)	1 (0.6%)	1 (0.2%)
Age (years)		61 (50–72)	57 (46–67)	60 (48–70)
Breslow depth (mm)		1.80 (1.30, 3.00)	1.90 (1.30, 3.20)	1.80 (1.30, 3.00)
Ulceration	Absent	261 (71.1%)	122 (72.6%)	383 (71.6%)
	Present	106 (28.9%)	39 (23.2%)	145 (27.1%)
	Unknown	0 (0%)	7 (4.2%)	7 (1.3%)
Disease stages *	IA	29 (7.9%)	10 (6.0%)	39 (7.3%)
	IB	156 (42.5%)	70 (41.7%)	226 (42.2%)
	IIA	108 (29.4%)	40 (23.8%)	148 (27.7%)
	IIB	44 (12.0%)	28 (16.7%)	72 (13.5%)
	IIC	30 (8.2%)	13 (7.7%)	43 (8.0%)
	Unknown	0 (0%)	7 (4.2%)	7 (1.3%)
Histologic type	SSM	180 (49.0%)	101 (60.1%)	281 (52.5%)
	NM	138 (37.6%)	50 (29.8%)	188 (35.1%)
	LMM	5 (1.4%)	1 (0.6%)	6 (1.1%)
	ALM	4 (1.1%)	3 (1.8%)	7 (1.3%)
	Other	40 (10.9%)	9 (5.4%)	49 (9.2%)
	Unknown	0 (0%)	4 (2.4%)	4 (0.7%)
Biopsy location	Head/Neck	0 (0%)	1 (0.6%)	1 (0.2%)
	Trunk	176 (48.0%)	80 (47.6%)	256 (47.9%)
	Upper extremities	80 (21.8%)	30 (17.9%)	110 (20.6%)
	Lower extremities	111 (30.2%)	56 (33.3%)	167 (31.2%)
	Unknown	0 (0%)	1 (0.6%)	1 (0.2%)
Clark level	II	17 (4.6%)	9 (5.4%)	26 (4.9%)
	III	169 (46.0%)	46 (27.4%)	215 (40.2%)
	IV	156 (42.5%)	58 (34.5%)	214 (40%)
	V	12 (3.3%)	6 (3.6%)	18 (3.4%)
	Unknown	13 (3.5%)	49 (29.2%)	62 (11.6%)
Angiolymphatic Invasion	Absent	0 (0%)	58 (34.5%)	58 (10.8%)
	Present	0 (0%)	6 (3.6%)	6 (1.1%)
	Unknown	367 (100%)	104 (61.9%)	471 (88.0%)

## Data Availability

Data are available upon reasonable request to the corresponding author and after approval of the scientific and privacy committees of PALGA and the NCR.

## Supplementary Materials

The following supporting information can be downloaded at: https://www.mdpi.com/article/10.3390/cancers14122854/s1, Table S1: A detailed overview of 5-year survival rates (RFS, DMFS, OS) in different (sub)stages in the European cohort (Sahlgrenska University Hospital, Erasmus MC Cancer Institute, and combined). Figure S1. Flowchart sample collection.
