# Supplementary material for: Using a Clinicopathologic and Gene Expression (CP-GEP) Model to Identify Stage I–II Melanoma Patients at Risk of Disease Relapse
Source: Cancers (Basel). 2022 Jun 9;14(12):2854. doi: 10.3390/cancers14122854 (PMC9220976; doi:10.3390/cancers14122854)
Supplement: Supplementary file 1 [file cancers-14-02854-s001.zip › cancers-1737272-supplementary.pdf]

Supplementary materials

Table S1. A detailed overview of 5-year survival rates (RFS, DMFS, OS) in different (sub)stages in the European cohort (Sahlgrenska University Hospital, Erasmus MC Cancer Institute, and combined). CP-GEP, clinicopathologic and gene expression profile; RFS, recurrence-free survival; SLNB, sentinel lymph node biopsy; 95%CI, 95% confidence interval.

**Sahlgrenska University Hospital**							
**Stratification**	***n***	**5 year RFS**	**95% CI**	**5 year DMFS**	**95% CI**	**5 year OS**	**95% CI**
Stage I-II	367	85%	(81-89)	90%	(86-92)	87%	(83-90)
Stage IA	29	100%	(100-100)	100%	(100-100)	97%	(78-100)
Stage IB	156	93%	(87-96)	97%	(92-99)	93%	(88-96)
Stage IIA	108	84%	(75-90)	88%	(79-93)	87%	(79-92)
Stage IIB	44	67%	(51-79)	76%	(60-86)	73%	(57-84)
Stage IIC	30	65%	(45-79)	68%	(48-82)	67%	(47-81)
**Erasmus MC Cancer Institute**							
**Stratification**	***n***	**5 year RFS**	**95% CI**	**5 year DMFS**	**95% CI**	**5 year OS**	**95% CI**
Stage I-II	168	79%	(71-85)	87%	(80-92)	86%	(79-91)
Stage IA	10	100%	(100-100)	100%	(100-100)	100%	(100-100)
Stage IB	70	94%	(81-98)	98%	(84-100)	91%	(79-97)
Stage IIA	40	80%	(63-90)	86%	(69-94)	89%	(72-96)
Stage IIB	28	42%	(19-64)	71%	(45-87)	77%	(50-91)
Stage IIC	13	42%	(16-67)	49%	(19-73)	67%	(34-86)
**Combined**							
**Stratification**	***n***	**5 year RFS**	**95% CI**	**5 year DMFS**	**95% CI**	**5 year OS**	**95% CI**
Stage I-II	535	84%	(80-87)	89%	(86-91)	87%	(83-89)
Stage IA	39	100%	(100-100)	100%	(100-100)	97%	(81-100)
Stage IB	226	93%	(89-96)	97%	(94-99)	93%	(88-96)
Stage IIA	148	83%	(76-88)	87%	(80-92)	87%	(81-92)
Stage IIB	72	60%	(46-71)	74%	(61-84)	74%	(61-83)
Stage IIC	43	58%	(41-72)	63%	(46-76)	66%	(50-79)

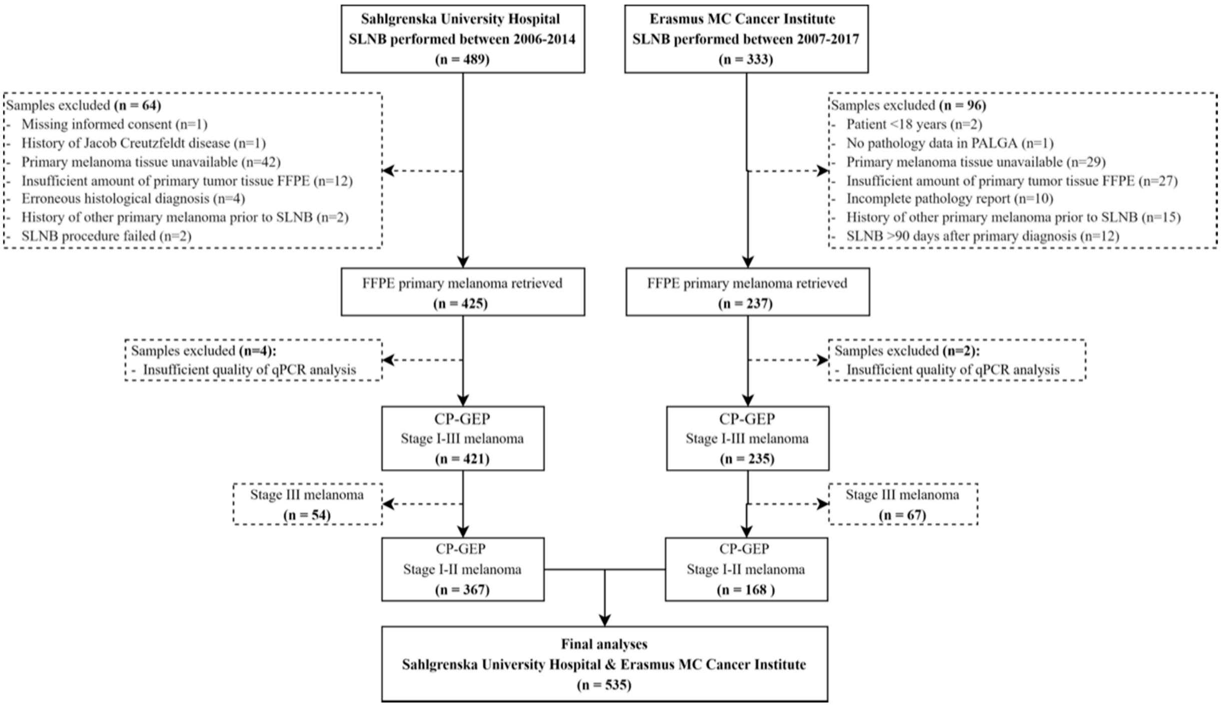

Figure S1. Flowchart sample collection. FFPE, formalin-fixed paraffin-embedded; MC, medical center; PALGA, Nationwide Network and Registry of Histopathology and Cytopathology; qPCR, quantitative polymerase chain reaction; SLNB, sentinel lymph node biopsy.
